# Evaluation of methods for linking household and health care provider data to estimate effective coverage of management of child illness: results of a pilot study in Southern Province, Zambia

**DOI:** 10.7189/jogh.08.010607

**Published:** 2018-06

**Authors:** Emily D Carter, Micky Ndhlovu, Thomas P Eisele, Emmy Nkhama, Joanne Katz, Melinda Munos

**Affiliations:** 1 Institute for International Programs, Johns Hopkins Bloomberg School of Public Health, Baltimore, Maryland, USA; 2 Chainama College of Health Sciences, Great East Road, Lusaka, Zambia; 3 Center for Applied Malaria Research and Evaluation (CAMRE), Tulane School of Public Health and Tropical Medicine, New Orleans, Louisiana, USA; 4 Department of International Health, Johns Hopkins Bloomberg School of Public Health, Baltimore, Maryland, USA

## Abstract

**Background:**

Existing population-based surveys have limited accuracy for estimating the coverage and quality of management of child illness. Linking household survey data with health care provider assessments has been proposed as a means of generating more informative population-level estimates of effective coverage, but methodological issues need to be addressed.

**Methods:**

A 2016 survey estimated effective coverage of management of child illness in Southern Province, Zambia, using multiple methods for linking temporally and geographically proximate household and health care provider data. Mothers of children <5 years were surveyed about seeking care for child illness. Information on health care providers’ capacity to manage child illness, or structural quality, was assessed using case scenarios and a tool modeled on the WHO Service Availability and Readiness Assessment (SARA). Each sick child was assigned the structural quality score of their stated (exact-match) source of care. Effective coverage was calculated as the average structural quality experienced by all sick children. Children were also ecologically linked to providers using measures of geographic proximity, with and without data on non-facility providers, to assess the effects of these linking methods on effective coverage estimates.

**Results:**

Data were collected on 83 providers and 385 children with fever, diarrhea, and/or symptoms of ARI in the preceding 2 weeks. Most children sought care from government facilities or community-based agents (CBAs). Effective coverage of management of child illness estimated through exact-match linking was approximately 15-points lower in each stratum than coverage of seeking skilled care due to providers’ limited structural quality. Estimates generated using most measures of geographic proximity were similar to the exact-match estimate, with the exception of the kernel density estimation method in the urban area. Estimates of coverage in rural areas were greatly reduced across all methods using facility-only data if seeking care from CBAs was treated as unskilled care.

**Conclusions:**

Linking household and provider data may generate more informative estimates of effective coverage of management of child illness. Ecological linking with provider data on a sample of all skilled providers may be as effective as exact-match linking in areas with low variation in structural quality within a provider category or minimal provider bypassing.

There are numerous proven interventions for preventing and managing child illness in low- and middle-income countries (LMICs). Despite the efficacy of these interventions, low intervention coverage and poor quality of care has limited their impact on child survival. Intervention coverage is defined as the proportion of a population in need of an intervention that receives the intervention [1]. Governments and other organizations implementing health programs need accurate and timely coverage data to improve or develop more effective child health programs and policies that accelerate reductions in child mortality.

Existing population-based household survey methods have limited accuracy for estimating the coverage of management of child illness due to issues including maternal recall, reporting bias, and disease identification [2-4]. Additionally, traditional coverage measures assess intervention need and utilization but do not typically account for quality of intervention delivery. There is growing interest in measures of “effective coverage,” which combine intervention need, utilization, and service quality, in monitoring progress towards universal health coverage [5]. The Improving Coverage Measurement Group has proposed linking information on the care-seeking collected through a household survey with assessments of the quality of services from facility or provider surveys as a means of generating more informative population-level estimates of the coverage of key health interventions [1]. Linking household and provider data may provide a more accurate picture of the quality of care received from a provider while maintaining a population-representative sample through the household survey. However, more evidence is needed about how to link these data to obtain valid and representative measures of effective coverage.

There are several methodological issues to consider when linking household and health care provider assessments. Linking analyses use information on care-seeking from household surveys to assign an individual to one or more potential sources of care to assess the service environment most likely encountered by individuals seeking care. A recent systematic review found almost 60 studies published since 1990 have linked information from household surveys and service environment assessments to address questions about access to and use of reproductive, maternal, newborn, and child health (RMNCH) interventions in LMICs [6]. The linking methodology and sources of household and provider data varied greatly across studies, each presenting unique issues including temporal and geographic disconnects in data sets, non-representative samples, and lack of information on all sources of care. Few studies employed “exact-match linking”, considered the most valid method for generating linked estimates, which assigns an individual to the specific provider(s) from which care was reportedly received [6]. The majority of studies performed “ecological linking” by assigning an individual or household to one or more health care provider(s) based on geographic proximity. Ecological linking may result in households or individuals being linked to a provider that may not accurately reflect the availability or quality of services accessed by an individual. However, ecological linking requires less arduous data collection than the alternative exact-match linking and may be a more feasible method for combining household and provider data at a large scale or in conjunction with existing global health data collection mechanisms. Most linking analyses utilize routinely collected national population-based surveys including the Demographic and Health Survey (DHS) and to a lesser extent Multiple Indicator Cluster Survey (MICS). Service Provision Assessments (SPAs) or Service Availability and Readiness Assessments (SARAs) are the most common sources of health care provider data for linking analyses [6]. These facility assessments collect data on only a sample of facility-based health care providers, limiting information on important sources of care including pharmacies, community-based and informal providers.

We implemented a study in Southern province, Zambia to assess the feasibility of collecting geographically and temporally concurrent household and health care provider data at a small scale in both an urban and rural setting to perform exact-match linking. We also aimed to quantify the degree of bias introduced by using less rigorous linking methods, including multiple ecological linking methods and utilization of facility-only health care provider assessments.

## METHODS

### Study site

The study was nested in a validation study of maternal reports of care-seeking for childhood illness [7] and was conducted in two urban and three rural health facility catchment areas in Choma District in Southern Province, Zambia, between January 18 and March 20, 2016. Choma district is primarily agrarian, although Choma town is a growing commercial hub and provincial capital [8]. Under five mortality rates in Zambia have declined dramatically over the past two decades; however, pneumonia, diarrhea, and malaria remain the leading causes of child mortality in the post-neonatal period [9].

Mothers report approximately 70 percent of children in Southern Province with fever, diarrhea, or ARI symptoms are taken for care, primarily in the public sector [9]. The Zambian government manages 90% of health facilities either directly or through service agreements with the Churches Health Association of Zambia (CHAZ). However, the private sector is growing in urban centers [10]. Health services are free for children <5 years at all government facilities, including hospitals with referral [11]. The Integrated Management of Child Illness (IMCI) approach has been implemented in all districts since the 1990s; however by the late 2000s only about 65% of health facilities had been staffed by an IMCI-trained clinician [12]. Community based health agents (CBAs) participate in task shifting at government facilities and implement a variable package of community-based interventions, including diagnosis and treatment of malaria and treatment of diarrhea with oral rehydration solution (ORS) [13]. The study area has been the site of ongoing malaria testing and treatment and mass drug administration trials [14,15].

### Study design, participants, and data collection

The study included two components; 1) a household survey on care-seeking for child illness, and 2) an assessment of health care providers’ structural quality for managing child illness. Ethical approval for the study was obtained from the Institutional Review Boards of Johns Hopkins Bloomberg School of Public Health and Excellence in Research Ethics and Science (ERES) Converge in Zambia. The Zambian Ministry of Health granted permission and the Choma District Health Office provided support to survey government health facilities in the study area.

In each of the urban and rural strata, 700 households were randomly sampled from the health facility catchment areas (HFCAs) of five government health facilities in and around Choma town. Households were randomly sampled from the catchment population of three rural health centers using an existing household listing created in 2014 [16]. Urban households were sampled from a census of households conducted immediately prior to the household enrollment phase. Households with a woman of reproductive age (15-49 years) with at least one biological child <59 m were eligible to participate in the study. These criteria correlate with the DHS requirements for the child questions in the Women’s Questionnaire and ensured that participating children were less than 5 years of age at the time of the household survey. A sample of 700 households per stratum was expected to yield information on 155 episodes of child illness per stratum, allowing estimation of effective coverage of management of child illness with a precision of ±6.0%, based on a type-1 error probability of 5% (two-tailed test) and an underlying standard deviation in care of 0.35.

Households were enrolled in the study from January 18 to February 13, 2016, and subsequently revisited approximately four to six weeks later for the household care-seeking survey completed between March 3-20, 2016. Mothers were asked about child illness and care-seeking using a questionnaire based on the 2013-2014 Zambia DHS (ZDHS). These included questions about the presence of diarrhea, fever, or suspected ARI in each of their children <5 years in the preceding two weeks. If a child experienced an illness, mothers were asked if care was sought, the source of care, and treatment received. In addition to the series of DHS care-seeking questions, mothers answered questions to ascertain the name of the specific source of care and sequence of care-seeking events. If the name of the source of care was unknown, data collectors were instructed to probe about provider location and other identifying features.

Concurrent to household enrollment, health care providers were identified and invited to participate in the provider assessment. The term health care provider will be used to refer to both individual providers such as CBAs and traditional practitioners, and health care outlets that include multiple staff such as health facilities and pharmacies. Public, private, informal, and traditional sources of care were included in the assessment. Community leaders and health workers initially provided a list of commonly utilized care providers offering medicine or alternative treatment for sick children. The list was further expanded with information from participating mothers about common sources of care for treating illness in their children <5 years collected during enrollment. All providers included in the assessment were grouped into categories of providers used in the ZDHS (**Box 1**) and this classification was employed in all ecological linking analyses restricted by provider category.

Box 1 **Categories of health care providers in the study area.**

Public

• Government hospital

• Government health center/post

• Government CBA / fieldworker

Private

• Private hospital/clinic

• Pharmacy

Informal

• Shop/market

• Traditional/faith-based practitioner

**Figure 1:**
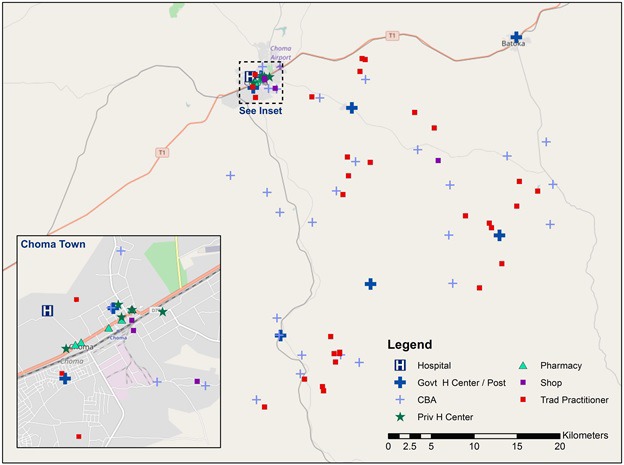
Map of health care provider locations.

The provider assessment was completed among all identified health care providers (**Figure 1**). The provider assessment was designed to assess a provider or facility’s capacity to provide curative services for children <5 years, including presence of drugs and commodities, training, supervision, and provider case management knowledge. The assessment was designed to assess a provider’s structural quality following the Donabedian structure-process-outcome model [17] and to align with the WHO definition of provider readiness [18] as upstream measures of health care provider quality. At facilities and pharmacies with multiple staff, the questionnaire was administered to the most senior staff member and reports of the existence and functionality of physical commodities (medicines, equipment, etc) were verified by observation. Questions were modeled off the SARA general and child health questionnaire [18] and adapted for use with facility-based, community-based, public, private, and informal providers. Clinical case scenarios developed for use in the evaluation of the IMCI program were used to assess provider case management knowledge [19]. Providers were read four clinical case scenarios and asked how they would manage each hypothetical sick child. At outlets with multiple clinical staff, up to three staff members within each cadre of clinical health workers were randomly selected among those available at the time of the assessment to respond to case scenarios.

### Analysis

#### Provider assessment scores

The provider assessment was used to generate a “structural quality score” corresponding to a provider’s structure or capacity to appropriately manage a child illness. The structural quality score measured availability of services, commodities, and human resources needed to appropriately manage common child illnesses (**Box 2**). These indicators were considered the minimum inputs for appropriate care: the basic commodities required to diagnosis and treat common child illness, along with the human resources and clinical knowledge to apply them correctly. As such, the score reflects an upper threshold of the potential quality of care offered by a provider. A provider received one point for each indicator if requirements were met and zero if not; each domain received equal weight. The knowledge domain was calculated as an average score of provider performance on four case scenarios. An average facility knowledge score was generated when knowledge was assessed for multiple health workers at a single facility. Providers were assessed against the expected capacity for their specific provider category; for example, CBAs were not penalized for not having antibiotics in this setting where CBAs are not allowed to treat ARI.

Box 2 **Structural quality score components**

Diagnostics

• Malaria Diagnostic (RDTs or microscopy)

• Malnutrition Diagnostic (MUAC or Scale + Height board + Growth chart)

• ARI Diagnostic (stethoscope or respiratory timer)

• General microscopy (functioning microscope and slides)

Basic medicines

• Oral rehydration solution

• Zinc

• Artemisinin combination therapy (ACT)

• Oral antibiotic

Severe/complicated illness medicines

• IV fluids

• Injectable quinine or artesunate

• Injectable antibiotics

Human Resources

• Training (at least one staff member with IMCI or relevant training)

• Guidelines (IMCI guidelines or relevant guidelines or job aid available)

• Supervision (received supervision visit with case management observation in past 3 months)

Available services

• Diagnosis and treat malaria (by pathology)

• Diagnosis and treat diarrhea (by pathology)

• Diagnosis and treat ARI (by pathology)

• Diagnosis and treat malnutrition (by pathology)

• Facilitated referral capacity

Knowledge

• Average performance on case scenarios

#### Linking care-seeking and provider data

The primary outcome was input-based effective coverage of management of child illness estimated through exact-match linking and ecological linking methods. Effective coverage of management of child illness was calculated as the average level of structural quality experienced by sick children based on their reported care-seeking behavior and linked source of care. Each child was assigned the structural quality score of either their specific reported source(s) of care (exact-match linking) or the closest provider(s) based on measures of geographic proximity (ecological linking). The linking was performed using provider assessment data on all health care providers, to reflect capacity among all categories of providers, and data on only health facilities, to replicate the provider data available through common provider assessments such as the SPA or SARA. We considered estimates of effective coverage generated through the exact-match linking using data on all health care providers to be the most accurate linked coverage estimate. However, we did not assess the validity of the effective coverage estimates generated through the method against a true measure of how sick children in the study area were managed.The input-based effective coverage estimate reflects an upper limit on the proportion of children that could have been correctly managed. Estimates generated using the ecological linking methods and using data on only facility-based providers were compared against the exact-match all-provider coverage estimates to assess their population-level validity, or how closely they reproduced the exact-match all-provider estimates of effective coverage.

For exact-match linking, each sick child was linked to the specific source(s) of care from which care was sought, based on the name of the facility, outlet, or provider reported by the mother during the household survey.

**Figure 2:**
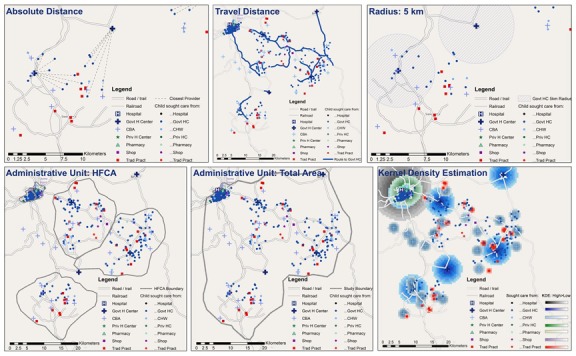
Illustration of ecological linking methods (household locations have been displaced in figure to protect confidentiality).

For ecological linking, each sick child was linked to the closest provider(s) based on various measures of geographic proximity. Seven methods for ecological linking were employed, depicted in Figure 2. Measures of geographic proximity employed in the ecological linking were adapted from the work of Skiles and colleagues [20]. Geographic proximity was calculated using ArcGIS 10.1 (Esri, Redlands, CA, USA). Specifications and steps for generating geographic links are presented in Appendix S1 in **Online Supplementary Document(Online Supplementary Document)**. Linked data sets were exported to Stata 14.2 (StataCorp LLC, College Station, TX, USA) for analysis.

#### Ecological linking methods

The seven measures of geographic proximity used in the ecological linking can be grouped into three categories: methods linking children 1) to the single nearest provider by distance, 2) to all providers within a defined geographic unit, and 3) using kernel density estimation.

Single nearest provider link:

- Nearest Absolute Distance: Child was linked to the single closest provider based on absolute distance within the reported source of care provider category. This is the simplest method for assigning a child to a specific provider.
- Nearest Travel Distance: Child was linked to the single closest provider by road distance within the reported source of care provider category. This method is designed to model the effect of road access and quality on care-seeking.
  Aggregation of Providers within Geographic Unit Link:
- Radius - 5 Kilometer: Child was linked to all providers within the source of care provider category within a 5 km radius of the child’s home. This method is designed to approximate a 1-hour walking distance from a household to a provider in any direction.
- Administrative Unit – HFCA: Child was linked to all providers with the source of care provider category within the health facility catchment area (HFCA) in which the child resides. This method is designed to mimic the effect of using aggregate data at a small scale (enumeration area / cluster) and corresponds with a government health facility designated catchment area.
- Administrative Unit – Total Study Area: Child was linked to all providers with the source of care provider category included in the study. This method is designed to mimic the effect of using aggregate data as a medium scale (sub-district).

Kernal Density Estimation (KDE): The KDE method was designed to model the level of draw a provider exerts over households as they decide to seek care, based on distance decay and characteristics of the provider. KDE has been used as a means of modeling health care access [21] and service environment [20]. KDE can be used to model health care utilization in the absence of household data assuming all individuals would seek care if skilled providers are accessible, and the choice of provider is driven by provider quality and distance. KDE employs a user-specified kernel size and probability density distribution. The kernel size, or maximum radius of a provider catchment area, was selected to reflect a household preference for higher-level providers. Higher-level providers (hospitals and health facilities) had a larger catchment area than lower level providers (pharmacies and community-based providers). Within a catchment area, a provider’s draw decreased with increasing distance from a household. A provider’s structural quality score was used as the density variable, effectively modeling higher draw within their catchment area for providers with higher scores. Each child was linked exclusively or partially to a category of provider based on the level of draw exhibited by providers in the category. Information on source of care from the household survey was excluded because the method models care-seeking behavior.

- KDE – Single Highest: Child was linked to the closest provider within the single provider category that exerted the greatest draw on the child’s household.
- KDE – Weighted Aggregate: Child was linked to the closest provider in all provider categories that exerted any draw on the household. The contributions of the linked providers’ structural quality scores were weighted by the draw for the provider category relative to the other provider categories.

#### Calculating effective coverage using all provider data

For the exact-match linking, each sick child was assigned the structural quality score for the specific source(s) from which care was sought. If care was reportedly sought from more than one source, the child was assigned the average score for all providers from which care was sought. If no care was sought for the illness, the child was assigned a structural quality score of zero. If the mother reported a source of care that could not be identified or included in the provider assessment, the child was assigned the average structural quality score for the source of care provider category within the stratum (urban/rural). Effective coverage was calculated as the average structural quality score (a percentage ranging from 0 to 100) across all sick children, including those who were not taken for care (who received a score of 0).

For the ecological linking, each sick child was assigned the structural quality score for the source(s) of care that were closest based on various measures of geographic proximity, as described above. All non-KDE ecological linking methods maintained the reported category of source of care. In other words, a child could not be linked to a provider from a category of source of care other than the category reported by the mother (eg, a child that was reported to have been taken to a government CBA could only be linked to government CBAs, although the specific CBA(s) to which he/she was linked might vary depending on the measure of geographic proximity). Similar to the exact-match linking, children that were not taken for care were assigned a structural quality score of zero. Those that could not be linked to a provider from the reported source of care were assigned the average structural quality score for the category of source of care within the stratum. For example, when applying the 5 km radius linking approach, if a rural mother reported that her child was taken to a government health center for care but there was no government health center within 5 km of the household, the child was assigned the average of all government health centers in the rural area. If a child was linked to multiple providers, the average structural quality score for all linked providers was calculated for the child. Effective coverage was calculated as the average structural quality score (a percentage ranging from 0 to 100) across all sick children, including those who were not taken for care (who received a score of 0).

#### Calculating effective coverage using facility-only provider data

To simulate the type of provider data that would typically be available when using a SPA or SARA for linking, we repeated the analysis using only facility data. Coverage was estimated using the exact-match linking method and each of the seven ecological linking methods with only facility structural quality scores. Health facilities were defined as either a government or private clinic or hospital, in line with those providers included in the SARA and SPA surveys. Presuming first-level government facilities offered the most comparable level of structural quality to government CBAs, children that reported care from a CBA were linked to one or more government health centers using the nearest provider and aggregate ecological linking methods, and assigned the average government health center structural quality score for the exact-match linking. Using the exact-match, nearest provider, and aggregate ecological linking methods, children who recieved treatment from all other sources of care (pharmacies, shops, and traditional practitioners) were treated as unskilled sources of care and assigned a structural quality score of zero, equivalent to seeking no care. All other components of the linking methodology and household data remained the same. Using the KDE methods, data on non-facility providers were excluded while modeling care-seeking behavior. A summary of all linking methods employed in the paper is presented in **Table 1**.

**Table 1:** Linking method summary

Linking method	Provider assessment data	
	**All providers**	**Facility-based providers only**
**Exact-match**	Link Sick Child to: Specific source of care (name) reported by mother	Link Sick Child to: Specific source of care (name) reported by mother if facility-based provider*
**Single match:**		
Nearest – absolute distance	Closest provider within reported source of care category	Closest provider in reported source of care category if facility-based provider*
Nearest – road distance	Closest provider by road distance within reported source of care category	Closest provider by road distance in reported source of care category if facility-based provider*
**Aggregate match:**		
Radius – 5 km	All providers within 5 km radius of home in reported source of care category	All providers within 5km radius of home in reported source of care category if facility-based provider*
Administrative unit – HFCA	All providers within HFCA in reported source of care category	All providers within HFCA in reported source of care category if facility-based provider*
Administrative unit – total area	All providers in study area in reported source of care category	All providers in study area in reported source of care category if facility-based provider*
**Kernel Density Estimation (KDE):**		
Single highest	Closest provider within single category of care exerting greatest KDE pull on household	Closest provider within single category of care exerting greatest KDE pull on household among facility-based providers
Weighted aggregate	Closest provider within all categories of care exerting KDE pull on household	Closest provider within all categories of care exerting KDE pull on household among facility-based providers

HFCA – health facility catchment area

*Children reporting seeking care from CBAs were linked 1) to government health centers (primary analysis – **Table 3** or **Table 2**) treated as no care (Table S9b in **Online Supplementary Document(Online Supplementary Document)**) during facility-only analyses. All other non-facility providers treated as no care and not linked.

Descriptive statistics comparing the ecological and facility-only links to the exact-match all provider links were calculated. Sensitivity analyses were also conducted to estimate effective coverage using different assumptions for children that could not be linked to a provider within the reported source of care category including: 1) assigning children that could not be linked to a provider based on geographic proximity a structural quality score of zero, and 2) assigning children that sought care from a CBA a score of zero during the facility-only analysis. These sensitivity analyses were designed to mimic the effect of service environment assessments and linking analyses that define health care access based on a capped maximum distance from a household and that ignore the contribution of CBAs in management of child illness, respectively.

## RESULTS

A total of 335 rural and 469 urban households with at least one eligible mother of a child <5 years were enrolled in the study. At the time of the household care-seeking survey, 43 households (5.3%) were unavailable to complete the survey because the participating mother(s) had moved outside of the study area or to an unknown residence for the remainder of the study period, and 14 households withdrew consent for the care-seeking survey. This resulted in a loss-to-follow-up of 7.1% of households.

Characteristics of participating children and mothers available at follow-up and included in the household care-seeking survey are shown in Table S1 in **Online Supplementary Document(Online Supplementary Document)**. Among the 1084 children included in the household survey, 35% of urban children and 36% of rural children experienced at least one illness meeting DHS criteria in the 2 weeks preceding the survey (**Table 2**). Fever was the most commonly experienced symptom in both the rural and urban areas. Mothers reported care was sought for 79% of rural children and 67% of urban children with an illness. Reported care-seeking from more than one source was uncommon. The majority of children sought care from a skilled provider. Government health centers were the primary reported source of care in both the urban (60%) and rural (61%) areas (**Table 2**). In the rural area, 18% of children were taken to a CBA for care. A small number of children were taken to shops and traditional practitioners in the rural area. In the urban area, care was sought for 5% of children from informal shops. Hospitals, pharmacies, and private facilities accounted for a small number of care-seeking events in the urban area.

**Table 2:** Characteristics of reported child illness and care-seeking events, by stratum

	Rural			Urban		
	**n = 547**	**%**	**95% CI**	**N = 537**	**%**	**95% CI**
**Proportion of children with at least one DHS illness**	199	36.4	32.4-40.5	186	34.6	30.7-38.8
**Reported child illness:**	199			186		
Diarrhea	23	11.6	7.8-16.8	50	26.9	21.0-33.7
Fever	117	58.8	51.8-65.4	85	45.7	38.7-52.9
ARI*	6	3	1.4-6.6	3	1.6	0.5-4.9
Diarrhea & Fever	28	14.1	9.9-19.6	35	18.8	13.8-25.1
Diarrhea & ARI	3	1.5	0.5-4.6	0	0	–
Fever & ARI	17	8.5	5.4-13.3	10	5.4	2.9-9.7
Diarrhea, Fever, & ARI	5	2.5	1.0-5.9	3	1.6	0.5-4.9
**Proportion of illnesses for which mother reported seeking care from:**	199			186		
Any provider	157	78.9	72.7-84.0	124	66.7	59.6-73.1
Skilled provider†	151	75.9	69.5-81.3	116	62.4	55.2-69.0
>1 provider	9	4.5	2.4-8.5	5	2.7	1.1-6.3
**Proportion of children that sought care from category of provider**‡:	199			186		
Government hospital	0	0	–	5	2.7	0.9-6.2
Government health center/post	122	61.3	54.2-68.1	111	59.7	52.3-66.8
Government CBA/fieldworker	36	18.1	13.0-24.2	1	0.5	0.0-3.0
Private hospital/clinic	0	0	-	1	0.5	0.0-3.0
Pharmacy	1	0.5	0.0-2.8	2	1.1	0.1-3.8
Shop/market	2	1	0.1-3.6	9	4.8	2.2-9.0
Traditional/faith-based practitioner	5	2.5	0.8-5.8	0	0	–

DHS – Demographic Health Survey, CBA – community-based agents, ARI – acute respiratory infection, CI – confidence interval

*ARI defined as cough with chest-related difficulty breathing.

†Skilled providers included government and private health facilities and government CBAs.

‡Calculated among all sick children – some children taken to multiple sources of care.

### Provider structural quality

**Figure 3:**
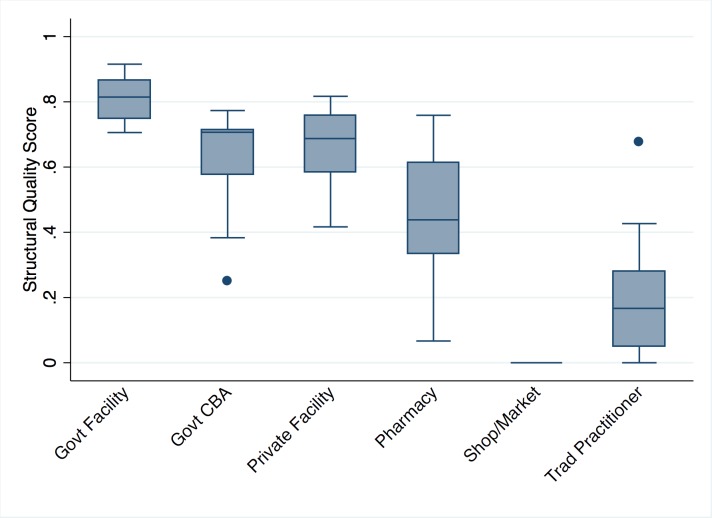
Median and interquartile range (IQR) of structural quality scores by provider category. *Presented using collapsed provider categories to preserve confidentiality of providers. Categories defined in **Box 1** used in all linking analyses restricted by source of care provider category.

All public and private facilities, pharmacies, and government CBAs offering child curative services, and the most commonly utilized traditional practitioners and informal drug outlets in the study area were included in the provider assessment for a total of 83 providers (Table S1 in **Online Supplementary Document(Online Supplementary Document)**). Government health facilities had the highest structural quality scores, followed by CBAs, private facilities, and pharmacies (**Figure 3**). Performance on each score domain by provider category is presented in Table S2 in **Online Supplementary Document(Online Supplementary Document)**. Facilities performed the poorest on domains related to training and guidelines and knowledge. CBAs performed poorly on availability of basic medicines and management capacity. Pharmacies excelled on measures of medicine availability but failed on all other measures. Shops and traditional practitioners performed poorly on all domains. **Figure 3** and Table S2 in **Online Supplementary Document(Online Supplementary Document)** present structural quality scores using collapsed provider categories to preserve anonymity for providers in small categories, however these values are descriptive and do not reflect the category-specific averages used in the aggregate linking approaches. Structural quality scores varied most by category of provider, and in most cases did not vary greatly within provider categories used in the aggregate linking (Table S3 in **Online Supplementary Document(Online Supplementary Document)**). While there were a few providers whose scores were notably above or below others within their category, these were provider categories that did not align with common source of care categories.

### Exact-match linking

Almost all mothers were able to report the name of the specific source from which care was sought for their sick child. All identified providers were included in the provider assessment, including 99% of rural care-seeking events and 93% of urban care-seeking events (**Table 3**). During the household survey, mothers reported eleven care-seeking events (2 rural, 9 urban) with providers, primarily shops, that could not be identified because they could not be named, sufficiently described for identification, or located for inclusion in the study. These children were assigned the average structural quality score for the category of source of care and strata.

**Table 3:** Proportion of sick children linked to provider from reported source of care category, by stratum and linking method

Linking method	Rural		Urban	
	**Number linked**	**% linked**	**# Linked**	**% linked**
**All providers:**	166		129	
**Exact-match**	164	98.8	120	93
**Single match:**				
Nearest - absolute distance	166	100	129	100
Nearest - road distance	166	100	129	100
**Aggregate match:**				
Radius – 5 km	106	63.8	129	100
Administrative unit – HFCA	165	99.4	129	100
Administrative unit – total area	166	100	129	100
**Facility-based providers only:**				
**Linking method**	166		129	
**Exact-match**	122	73.5	117	91
**Single match:**				
Nearest – absolute distance	122	73.5	117	91
Nearest – road distance	122	73.5	117	91
**Aggregate match:**				
Radius – 5 km	65	39	117	91
Administrative unit – HFCA	122	73.5	117	91
Administrative unit – total Area	122	73.5	117	91

HFCA – health facility catchment area

### Ecological linking

Using the nearest absolute distance and nearest travel distance linking methods, all children for whom care was sought were linked to a provider (**Table 3**). Most children taken for care were linked to their specific reported source(s) of care using the nearest absolute distance (89% rural, 88% urban) and nearest by travel distance (78% rural, 77% urban) methods (Table S4 in **Online Supplementary Document(Online Supplementary Document)**). This suggests that bypassing within a category of provider was low and absolute distance was a better measure for approximating the true source of care in this setting. Among those children taken to a more distant provider by absolute distance, there was no evidence of systematically bypassing poorer performing providers for better quality providers within the same provider category (data not shown).

Using the 5 km radius measure, greater than 35% of rural children that sought care were not linked to a provider because they resided more than 5 km from any provider within the reported category of source of care and were assigned the average structural quality score for the category of source of care (**Table 3**). This included almost half of rural children that sought care from a government health center (Table S5 in **Online Supplementary Document(Online Supplementary Document)**). In this setting, rural children on average travelled 5.4 km, up to a maximum of 16 km, to access a rural government facility (Table S6 in **Online Supplementary Document(Online Supplementary Document)**). However, all children that sought care in the urban area were linked to at least one provider because of the high density of providers. The total area boundary measure linked all children that sought care to at least one provider while the HFCA boundary measure linked all children with the exception of one child that sought treatment from a pharmacy in a rural catchment area with no pharmacies. The average number of providers to which a child linked by aggregate linking method is shown in Table S7 in **Online Supplementary Document(Online Supplementary Document)**.

Using the KDE methods, all urban children and 80% of rural children were linked to at least one source of care, representing a higher care-seeking rate than observed in the study (Table S8 in **Online Supplementary Document(Online Supplementary Document)**). The KDE single link method linked 70% of rural children and 84% of urban children to a skilled care provider. The KDE weighted link method linked 73% of rural children and 100% of urban children to a skilled care provider. The KDE single link method reproduced the care-seeking behavior of almost 40% of rural children but only 1% of urban children (data not shown).

### Effective coverage of management of child illness

Based on the household survey, mothers reported a high proportion of sick children were taken to a skilled provider for care, including 76% (95% CI = 70 – 81) of rural children and 62% (95% CI = 55-69) of urban children. Using the exact-match linking method, effective coverage of management of child illness was estimated at 60% (95% CI = 56-65) in the rural area and 49% (95% CI = 44-54) in the urban area (**Table 4**). The 16-point rural gap and 13-point urban gap in coverage between seeking skilled care and effective coverage was attributable to health care providers’ less than adequate capacity to manage child illness. Estimated effective coverage by structural quality domain is presented in **Table 5**. In the rural area, inconsistent stocks of medicines for managing complex or severe disease and low provider knowledge for managing child illness reduced overall estimates of effective coverage of management of child illness. In the urban area, low provider knowledge and inconsistent provider training, supervision, and access to job aids limited effective coverage.

**Table 4:** Effective coverage of management of child illness and difference in estimate from the exact-match all provider coverage, by linking method and stratum

Linking method	Rural				Urban			
	**%**	**95% CI**	**Diff**	***P***	**%**	**95% CI**	**Diff**	***P***
**All providers:**								
**Exact-match**	60.3	55.6-65.1	REF		49	43.6-54.5	REF	
**Single match:**								
Nearest – absolute distance	61.1	56.3-65.9	0.8	>0.05	49.1	43.7-54.6	0.1	>0.05
Nearest – road distance	58.8	54.1-63.5	-1.5	>0.05	48.7	43.2-54.1	-0.3	>0.05
**Aggregate match:**								
Radius – 5 km	59.4	54.8-64.1	-0.9	>0.05	49.2	43.7-54.7	0.2	>0.05
Administrative unit – HFCA	59.8	55.1-64.5	-0.5	>0.05	49.1	43.6-54.6	0.1	>0.05
Administrative unit – total area	57.9	53.4-62.4	-2.4	>0.05	49.4	43.9-54.9	0.4	>0.05
**KDE:**								
Single highest	55	50.4-59.6	-5.3	<0.05	71.8	69.3-74.2	22.8	<0.001
Weighted aggregate	54.9	50.4-59.5	-5.4	<0.05	74.3	73.2-75.5	25.3	<0.001
**Facility-based providers only:**								
**Exact-match**	62.1	57.1-67.0	1.8	>0.05	48.7	43.2-54.2	-0.3	>0.05
**Single match:**								
Nearest-absolute distance	62.6	57.6-67.6	2.3	>0.05	48.7	43.2-54.2	-0.3	>0.05
Nearest-road distance	61	56.2-65.9	0.7	>0.05	48.6	43.1-54.0	-0.4	>0.05
**Aggregate match:**								
Radius – 5 km	61.2	56.4-66.1	0.9	>0.05	48.9	43.4-54.5	-0.1	>0.05
Administrative unit – HFCA	62.8	57.8-67.8	2.5	>0.05	48.8	43.3-54.3	-0.2	>0.05
Administrative unit – total area	59.9	55.2-64.6	-0.4	>0.05	49	43.5-54.6	0	>0.05
**KDE:**								
Single highest	38.6	32.8-44.4	-21.7	<0.001	79	77.8-80.3	30	<0.001
Weighted aggregate	38.6	32.8-44.4	-21.7	<0.001	82.4	81.9-82.9	33.4	<0.001

CI – confidence interval, Diff – difference, KDE – kernel density estimation, HFCA – health facility catchment area

**Table 5:** Coverage of care-seeking and appropriate management of childhood illness by structural quality domain estimated through exact-match all provider linking, by stratum

Measure	Rural		Urban	
	**%**	**95% CI**	**%**	**95% CI**
**Sought care**	78.9	72.7-84.0	66.7	59.6-73.1
**Skilled provider**	75.9	69.5-81.3	62.4	55.2-69.0
**Structural quality score domain:**				
Diagnosis	66.7	61.0-72.4	61.8	54.9-68.8
Basic medicines	66.5	60.5-72.5	51	45.2-56.8
Complex medicines	48.3	42.6-54.0	57.2	50.6-63.8
Human resources	58	52.8-63.3	24.7	21.4-27.9
Capacity	66.9	61.1-72.8	61.2	54.3-68.1
Knowledge	43.6	39.6-47.5	36.9	32.6-41.1
**Effective coverage of management of child illness**	60.3	55.6-65.1	49	43.6-54.5

CI – confidence interval

The single nearest provider and aggregation of providers by geographic unit linking methods generated similar effective coverage estimates to the exact-match linking method (**Table 4**). None of the effective coverage estimates were statistically different from the exact-match effective coverage estimate, falling within ±3 percentage points of the exact-match point estimate by stratum. Despite failing to link a large proportion of rural children to a provider, the 5 km radius method generated similar coverage estimates when unlinked children were assigned the average provider category score. However, when unlinked cases were treated as having received no care, the 5 km radius method significantly underestimated coverage of appropriate care in the rural area (Table S9a in **Online Supplementary Document(Online Supplementary Document)**). Both KDE linking methods generated similar estimates of effective coverage in the rural area (55%) but overestimated effective coverage in the urban area (KDE single link: 72%, KDE weighted link: 74%) by overestimating the proportion of sick children taken for care.

### Facility-only linking

Exact-match linking and ecological linking were also performed using only health facility data. Using the exact match linking method, the proportion of rural children linked to their specific reported source of care was reduced from 99% to 74% (**Table 3**), due to exclusion of CBAs. However, the proportion of children linked to their specific provider remained similar among urban children.

Use of facility-only data further reduced the number of children in the rural area that were linked to their specific reported source of care through the single link ecological methods (68% absolute nearest, 61% travel distance) by excluding care-seeking events with CBAs and other community-based providers (Table S4 in **Online Supplementary Document(Online Supplementary Document)**). Under the 5 km radius linking method, the proportion of rural children that could not be linked to a provider from the reported source category increased to 61%, while the proportion of urban children that could not be linked remained low at 9% (**Table 3**). The proportion of children not linked using the HFCA and total area aggregate methods mirrored the exact-match linking method. Using the KDE methods, links to non-facility based providers were dropped, increasing the number of children linked to public and private providers with higher structural quality scores (Table S8 in **Online Supplementary Document(Online Supplementary Document)**).

**Figure 4:**
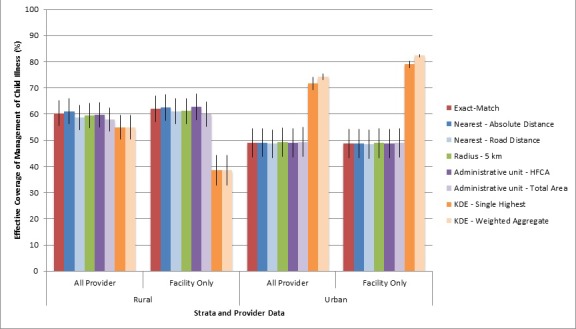
Effective coverage of management of child illness by linking method and stratum.

Estimates of effective coverage of management of child illness calculated using facility-only data did not differ significantly from the all-provider estimates using the exact-match, single nearest provider, and aggregation of providers by geographic unit linking methods when CBA care-seeking events were linked to government health centers (**Table 4**). None of the coverage estimates were statistically different from the exact-match effective coverage estimate calculated using data on all providers. Coverage estimated using the KDE weighted link increased due to the removal of partial links to providers with lower structural quality scores (pharmacies, traditional practitioners, etc.). Variation in effective coverage estimates generated using exact-match linking, ecological linking, and facility-only data are shown in **Figure 4**. If CBAs, a source of care for 18% of sick rural children, were treated as an unskilled source of care and given a structural quality score of zero, estimates of effective coverage in the rural area were greatly reduced across all linking methods (Table S9b in **Online Supplementary Document(Online Supplementary Document)**). The difference in the facility-only estimates from the exact-match all-provider estimates ranged from -9 to -22 percentage points in the rural area if CBAs were treated as unskilled providers during the linking analysis.

## DISCUSSION

We compared the estimated exact-match input-based effective coverage of management of child illness, considered our most accurate linked estimates, to estimates generated through ecological linking and use of facility-only provider data to assess the population validity of these linking methods. Most mothers were able to report on their sick child’s specific source(s) of care, and structural quality data was collected on most sources of care in the study area. Effective coverage of management of child illness was approximately 15 percentage points lower than skilled care-seeking in the study area due to providers’ imperfect structural quality. All non-kernel density estimation ecological linking methods produced similar estimates to the exact-match linking when children who could not be linked to a provider based on geographic proximity were assigned an average provider category score. Linking using data only on health facilities, like that available through the SPA or SARA, produced similar estimates when first-level health facilities were used as proxies for CBAs. These findings suggest linking household and health care provider data may be a viable method for generating more informative estimates of management of child illness, and less rigorous methods may be as effective as exact-match linking in certain contexts.

We found that most mothers could report their sick child’s specific source of care and structural quality data could be collected on the majority of health care providers in the study area, making the exact-match linking method feasible at this small scale. Almost all care-seeking events took place with a provider included in the provider assessment. The method failed to identify some minor sources of care, primarily informal shops in the urban area. Informal shops had very low potential for appropriate management of child illness, suggesting exclusion of these providers from the provider assessment may be justified. However, in settings with greater diversity in sources of care, ability to identify and assess private and community-based providers may be a limiting factor.

A number of analyses have used ecological linking methods to assess access to primary care or the effect of service environment on utilization of curative services including KDE [20], administrative unit [22,23], travel [24–26] and absolute distance [25,27]. However, none assessed the validity of ecological links against a measure of specific source of care. In this setting, many of the ecological linking methods closely estimated the exact-match linking results. In this study area where the majority of sick children were taken to the closest government health center for care, nearest provider linking methods with a census of providers were able to effectively reproduce this behavior and approximate exact-match estimates of coverage. Additionally, low variation in structural quality scores within commonly utilized categories of providers and little evidence of bypassing poorer quality sources within a provider category meant methods that linked children to an aggregation of providers within the category of care produced similar estimates to the nearest provider and exact-match linking methods. However, these methods may be inappropriate in settings with greater variation in quality or higher frequency of bypassing within provider categories, or less narrowly defined aggregation categories.

Ecological linking methods did present some limitations. In areas with a low density of providers, ecological linking methods such as the 5 km radius method that cap the maximum link distance between a household and provider may fail to link children to a source of care. Conversely, KDE methods may overestimate care-seeking behavior and subsequently effective coverage in areas with a high density of skilled providers due to an overestimation of the draw exerted by providers. KDE was ineffective in modeling care-seeking behaviors in this context. Collecting household data on the category of provider from which care was sought and maintaining this information during linking analyses was important in generating accurate measures due to variation in structural quality between categories of providers. There was evidence in this population of bypassing lower-level providers, such as CBAs, for government health facilities, further emphasizing the need to collect and maintain information on source of care category.

The availability of provider data for all sources of care vs only health facilities can also affect estimates of coverage. CBAs are a skilled source of care for some child illnesses in Zambia [28]. We found that use of facility-only service assessment data, like that available through SPA and SARA surveys, significantly underestimated effective coverage of management in rural areas if CBAs were treated as unskilled providers. We expect that similar effects would be seen in other settings where community-based or non-health facility providers offer appropriate care and are an important source of care for sick children. In this setting, the structural quality of first-level health facilities served as a reasonable proxy of CBA quality, slightly over-estimating of the quality of services offered by CBAs. However, service environment data from facility-based provider categories may not be an accurate substitute for estimating quality offered by these non-facility providers in other settings and should be assessed empirically to determine if it is a reasonable proxy.

SPA and SARA surveys often only collect data on a sample of facilities. Our study was too small to explore the effect of sampling on coverage estimates, however, a study by Skiles and colleagues found ecological linking with a sample of facilities resulted in high misclassification of links to closest providers and inaccurate estimates of service environment when compared to a facility census [20].

The generalizability of these results is limited by its small scale and low diversity in sources of care for child illness. The public sector is an important source of care for child illness in many sub-Saharan African countries [29], however the study area had higher levels of public sector care-seeking compared to some other sub-Saharan African settings [30]. It is unclear how these methods would have performed in a provider landscape with greater rates of care-seeking from private sector or informal providers. Additionally, the study was conducted in a small geographic area. This limited our ability to assess variation in structural quality across a wider sample of providers, the potential effect of provider bypassing on ecological linking estimates, and the effect of using aggregate administrative unit linking at a district or provincial level. Finally, additional work is needed in the development of a valid measure predictive of the quality of management of a sick child. We used a measure based on commodity availability, training, supervision, and provider knowledge, indicators that have been used to assess provider capacity in multiple settings and have been found to be associated with quality health worker performance [19,31–40]. However, there is known gap between health worker capacity and knowledge, and health worker performance [31]. Ideally, estimates of effective coverage would use a measure of process quality (quality of assessment, diagnosis, treatment and counseling of the sick child) or quantitative health gain from intervention receipt to assess quality [41]. The fact that our measure did not include an assessment of provider performance or health outcomes is a limitation; however our results are useful in examining the effect of various linking methods on effective coverage estimates.

## CONCLUSIONS

Linking household and provider data may generate more informative estimates of effective coverage of management of child illness. Our results suggest ecological linking with provider quality data on at least a sample of all skilled providers may be as effective as exact-match linking in areas with low variation in structural quality within commonly utilized provider categories or minimal provider bypassing. Assessment of non-facility providers is important in areas where these providers are a significant source of skilled care for sick children. Additionally, ecological linking methods must maintain or effectively reproduce apportionment of source of care by category of provider. This methodology is promising because it utilizes existing data collection mechanisms to generate a more complete picture of the management of child illness. This study was conducted on a small scale in an area with high rates of care-seeking for child illness from the nearest public sector provider. More studies are needed at a larger scale and in areas with a more diverse health care provider landscape to further evaluate the feasibility of the linking methodology. Additional research will support the development of guidelines for conducting linking assessments and potentially integrating this methodology into existing data collection mechanisms.

## References

[1] Bryce J, Arnold F, Blanc A, Hancioglu A, Newby H, Requejo J (2013). Measuring coverage in MNCH: new findings, new strategies, and recommendations for action.. PLoS Med.

[2] Eisele TP, Silumbe K, Yukich J, Hamainza B, Keating J, Bennett A (2013). Measuring coverage in MNCH: accuracy of measuring diagnosis and treatment of childhood malaria from household surveys in Zambia.. PLoS Med.

[3] Hazir T, Begum K, el Arifeen S, Khan AM, Huque MH, Kazmi N (2013). Measuring coverage in MNCH: A prospective validation study in Pakistan and Bangladesh on measuring correct treatment of childhood pneumonia.. PLoS Med.

[4] Fischer Walker CL, Fontaine O, Black RE (2013). Measuring coverage in MNCH: current indicators for measuring coverage of diarrhea treatment interventions and opportunities for improvement.. PLoS Med.

[5] Boerma T, Eozenou P, Evans D, Evans T, Kieny MP, Wagstaff A (2014). Monitoring progress towards universal health coverage at country and global levels.. PLoS Med.

[6] Do M, Micah A, Brondi L, Campbell H, Marchant T, Eisele T (2016). Linking household and facility data for better coverage measures in reproductive, maternal, newborn, and child health care: systematic review.. J Glob Health.

[7] Carter ED, Ndhlovu M, Munos M, Nkhama E, Kazt J, Eisele TP (2018). Validity of maternal report of care-seeking for childhood illness.. J Glob Health.

[8] Republic of Zambia Ministry of Local Government and Housing. Southern Province 2016 Available: http://www.mlgh.gov.zm/?page_id=656. Accessed: 7 September 2016.

[9] Central Statistical Office (CSO) [Zambia], Ministry of Health (MOH) [Zambia], ICF International. Zambia Demographic and Health Survey 2013-2014. Rockville, Maryland, USA: Central Statistical Office, Ministry of Health, and ICF International; 2014.

[10] Ministry of Health (MOH) [Zambia]. National Health Strategic Plan 2011-2015. 2011. Available: http://www.moh.gov.zm/docs/nhsp.pdf. Accessed: 20 September 2016.

[11] World Health Organization African Health Observatory. Comprehensive Analytical Profile: Zambia. 2016. Available: http://www.aho.afro.who.int/profiles_information/index.php/Zambia:Index. Accessed: 21 September 2016.

[12] Ministry of Community Development. Mother and Child Health, Ministry of Health (MOH) [Zambia]. Roadmap for Accelerating Reduction of Maternal, Newborn and Child Mortality, 2013 - 2016. 2013. Available: http://www.mcdmch.gov.zm/sites/default/files/downloads/MNCH_Road%20Map.pdf. Accessed: 15 September 2016.

[13] UNICEF. Access to healthcare through community health workers in East and Southern Africa. 2014. Available: http://www.unicef.org/health/files/Access_to_healthcare_through_community_health_workers_in_East_and_Southern_Africa.pdf. Accessed: 15 September 2016.

[14] Larsen DA, Bennett A, Silumbe K, Hamainza B, Yukich JO, Keating J (2015). Population-wide malaria testing and treatment with rapid diagnostic tests and artemether-lumefantrine in southern Zambia: A community randomized step-wedge control trial design.. Am J Trop Med Hyg.

[15] Eisele TP, Bennett A, Silumbe K, Finn TP, Chalwe V, Kamuliwo M (2016). Short-term impact of mass drug administration with dihydroartemisinin plus piperaquine on malaria in southern Province Zambia: A cluster-randomized controlled trial.. J Infect Dis.

[16] Eisele TP, Silumbe K, Finn T, Chalwe V, Kamuliwo M, Hamainza B (2015). Assessing the effectiveness of household-level focal mass drug administration (fMDA) and community-wide mass drug administration (MDA) with dihydroartemisinin+ piperaquine for reducing malaria parasite infection prevalence and incidence in Southern Province, Zambia: study protocol for a randomized controlled trial.. Trials.

[17] Donabedian A (1966). Evaluating the quality of medical care.. Milbank Mem Fund Q.

[18] World Health Organization. USAID. Service Availability and Readiness Assessment (SARA) Reference Manual Version 2.2. Geneva: World Health Organization; 2015.

[19] Bryce J, Victora CG, Habicht JP, Vaughan JP, Black RE (2004). The multi-country evaluation of the integrated management of childhood illness strategy: Lessons for the evaluation of public health interventions.. Am J Public Health.

[20] Skiles MP, Burgert CR, Curtis SL, Spencer J (2013). Geographically linking population and facility surveys: methodological considerations.. Popul Health Metr.

[21] Spencer J, Angeles G (2007). Kernel density estimation as a technique for assessing availability of health services in Nicaragua.. Health Serv Outcomes Res Methodol.

[22] Acharya LB, Cleland J (2000). Maternal and child health services in rural Nepal: does access or quality matter more?. Health Policy Plan.

[23] Micah A. “If you build it, will they come?” Facility-Level Characteristics that Determine Demand for Health Care Services in Rural Uganda. ASHE 2014Available: https://ashecon.confex.com/ashecon/2014/webprogram/Paper2864.html. Accessed: 1 February 2018.

[24] Buor D (2005). Determinants of utilisation of health services by women in rural and urban areas in Ghana.. GeoJournal.

[25] Kruk ME, Rockers PC, Williams EH, Varpilah ST, Macauley R, Saydee G (2010). Availability of essential health services in post-conflict Liberia.. Bull World Health Organ.

[26] Tanser F, Gijsbertsen B, Herbst K (2006). Modelling and understanding primary health care accessibility and utilization in rural South Africa: an exploration using a geographical information system.. Soc Sci Med.

[27] Akin JS, Guilkey DK, Hutchinson PL, McIntosh MT (1998). Price elasticities of demand for curative health care with control for sample selectivity on endogenous illness: an analysis for Sri Lanka.. Health Econ.

[28] Hamer DH, Brooks ET, Semrau K, Pilingana P, MacLeod WB, Siazeele K (2012). Quality and safety of integrated community case management of malaria using rapid diagnostic tests and pneumonia by community health workers.. Pathog Glob Health.

[29] Winter R, Wang W, Florey L, Pullum T. Levels and trends in care seeking for childhood illness in USAID MCH priority countries. DHS Comparative Reports No. 38. Rockville, MD: ICF International; 2015.

[30] Geldsetzer P, Williams TC, Kirolos A, Mitchell S, Ratcliffe LA, Kohli-Lynch MK (2014). The recognition of and care seeking behaviour for childhood illness in developing countries: A systematic review.. PLoS One.

[31] Rowe AK, de Savigny D, Lanata CF, Victora CG (2005). How can we achieve and maintain high-quality performance of health workers in low-resource settings?. Lancet.

[32] Amaral J, Gouws E, Bryce J, Leite AJ, Cunha AL, Victora CG (2004). Effect of Integrated Management of Childhood Illness (IMCI) on health worker performance in Northeast-Brazil.. Cad Saude Publica.

[33] Gouws E, Bryce J, Habicht J-P, Amaral J, Pariyo G, Schellenberg JA (2004). Improving antimicrobial use among health workers in first-level facilities: results from the Multi-Country Evaluation of the Integrated Management of Childhood Illness strategy.. Bull World Health Organ.

[34] Pariyo GW, Gouws E, Bryce J, Burnham G, Uganda IMCI Impact Study Team (2005). Improving facility-based care for sick children in Uganda: training is not enough.. Health Policy Plan.

[35] Rowe AK, Onikpo F, Lama M, Deming MS (2012). Evaluating health worker performance in Benin using the simulated client method with real children.. Implement Sci.

[36] Rowe AK, Hamel MJ, Flanders WD, Doutizanga R, Ndoyo J, Deming MS (2000). Predictors of correct treatment of children with fever seen at outpatient health facillities in the Central African Republic.. Am J Epidemiol.

[37] Mutale W, Godfrey-Fausset P, Mwanamwenge MT, Kasese N, Chintu N, Balabanova D (2013). Measuring health system strengthening: Application of the balanced scorecard approach to rank the baseline performance of three rural districts in Zambia.. PLoS One.

[38] El Arifeen S, Blum LS, Hoque DE, Chowdhury EK, Khan R, Black RE (2004). Integrated Management of Childhood Illness (IMCI) in Bangladesh: early findings from a cluster-randomised study.. Lancet.

[39] Gouws E, Bryce J, Pariyo G, Armstrong Schellenberg J, Amaral J, Habicht JP (2005). Measuring the quality of child health care at first-level facilities.. Soc Sci Med.

[40] Zurovac D, Rowe AK, Ochola SA, Noor AM, Midia B, English M (2004). Predictors of the quality of health worker treatment practices for uncomplicated malaria at government health facilities in Kenya.. Int J Epidemiol.

[41] Ng M, Fullman N, Dieleman JL, Flaxman AD, Murray CJ, Lim SS (2014). Effective coverage: A metric for monitoring universal health coverage.. PLoS Med.

