# Supplementary material for: Evaluation of methods for linking household and health care provider data to estimate effective coverage of management of child illness: results of a pilot study in Southern Province, Zambia
Source: J Glob Health. 2018 Jun 21;8(1):010607. doi: 10.7189/jogh.08.010607 (PMC6013179; doi:10.7189/jogh.08.010607)
Supplement: Online Supplementary Document [file jogh-08-010607-s001.pdf]

Online Supplementary Document

Carter et al. Evaluation of methods for linking household and health care provider data to estimate effective coverage of management of child illness: results of a pilot study in Southern Province, Zambia

J Glob Health 2018;8:010607

Appendix S1

Geographic proximity was calculated using ArcGIS 10.1. The longitude and latitude of the location of households with sick children and healthcare providers were input as XY data in decimal degrees and converted to point features by applying a geographic coordinate system (WGS 1984). Location data were converted to a geodatabase with a planar system (2-dimensional Cartesian plane) by projecting the data frame to the appropriate planar coordinate system for Zambia (universal transverse Mercator (UTM) zone 35S). Separate shape files were generated for each category of provider. These features were used in calculating geographic proximity:

Nearest Absolute Distance: A household with a sick child was linked to the closest provider within the reported category of source of care using the Near Features tool. Household location was used as the input feature and the provider location (by category) was the near feature. The procedure generated a variable in the household attribute table of the ID for the closest provider by absolute distance. This procedures was repeated with each of the 7 categories of providers and the household locations of children that sought care from those provider categories.

Nearest Travel Distance: A household with sick child was linked to the closest provider by road using the Closest Facility Analysis tool within the Network Analyst suite. Road network information for the study area was imported from Open Street Maps (OSM). As only roads were included in the OSM file, large trails and dirt paths were manually added to the road network based on satelite imagery. Provider locations (by category) were classified as "facilities" and households were defined as "incidents." The analysis tool calculated the fastest route from each incident (household) to a facility (provider) along the road network. Distance from the household to the closest road was treated as zero. The analysis accounted for the quality of the road on potential routes to providers. The hierarchy attribute was used to weight preference for better quality roads – mimicking the effect of faster travel times and greater availability of vehicular transportation. Paved roads were given preference over graded dirt roads, and graded dirt roads were given preference over ungraded roads and walking trails. No additional barriers or restrictions were used, other than existing breaks in the road network. Time was defined as the cost attribute for the impedence, resulting in calculation of the route with the minimal time from houshold to provider. Data on the starting household and closest provider by road was stored in the Route attribute table. This procedure was repeated with each of the 7 categories of providers and the household locations of children that sought care from those provider categories.

Radius (5 Kilometer): A household with a sick child was linked to all providers within the source of care category within a 5 km radius of the child's home using the Buffer and Interect tool. The planar method was used to generate a Euclidean buffer around each household with a straight-line radius of 5 km. Household locations were used as the input feature with a buffer distance of 5 km. The Intersect tool was used to compute the geometric intersection of the resulting household buffer layer and the locations of providers. The household buffer polygon feature and the provider location point feature were used as the inputs. The resulting output feature attribute table identified all providers falling within the 5 km radius (intersecting the buffer) of each household. This procedure was repeated with each of the 7 categories of providers and the household locations of children that sought care from those provider categories.

KDE: A household with a sick child was linked to provider(s) exerting the strongest pull over distance weighted by structural quality score and provider category using the Kernel Density tool within the Spatial Analyst suite. KDE parameters were adapted from those employed by Skiles [21]. Kernel size was defined by provider category with higher-order facilities receiving a larger kernel size:

- Hospitals: 10 km radius
- Government health centers and private clinics : 5 km radius
- Government CBAs and pharmacies: 2 km radius
- Traditional practitioners and informal shops: 1 km radius

The location of providers (by category) was used as the input feature. The provider structural quality score was specified as the "population," or density, value. The search radius, or kernel size, varied by the provider category as specified above. The procedure generated an output raster with a cell size of 500 m. The "Extract values to points" function was then used to calculate the raster value at the point location of each household. The household attribute table then contained the weighted pull value exerted by the category of provider at the location of the household. This procedure was repeated with each of the 7 categories of providers and the household locations of children that sought care from those provider categories. Two methods were used to assign a child a structural quality score based on the "pull" values generated through the KDE:

- Each child was linked to the closest provider within the source of care category exerting the strongest pull.
- Each child was linked to the closest provider within all categories of source of care exerting any pull on the household. The structural quality score assigned to each child was weighted based on the level of draw exerted by the category of provider.

Relevant attribute tables for proximity measures generated in ArcGIS were exported as CSV files. The CSV was converted to a Stata data set and merged with the child illness household survey data set for analysis in Stata. Two additional measures of geographic proximity were generated in Stata 14.2:

Administrative Unit (HFCA): Each sick child was assigned the average structural quality score of all providers within the source of care category within the HFCA in which the household resides. The average structural quality score for all providers within a category in each HFCA was calculated. Each provider was assigned to an HFCA based on their location within facility catchment boundaries. This average score was then linked to each sick child based on the HFCA of the household location and the

reported category of source of care (e.g. a sick child in Mochipapa HFCA treated by a CBA was assigned an average structural quality score of all CBAs within Mochipapa HFCA).

Administrative Unit (Study Area): Each sick child was assigned the average structural quality score of all providers within the source of care category within the total study area. The average structural quality score for all providers within a category was calculated. This average score was then linked to each sick child based on the reported category of source of care (e.g. a sick child in Mochipapa HFCA treated by a CBA was assigned an average structural quality score of all CBAs within the total study area).

Supplementary Table 1. Characteristics of participating children, mothers, households and healthcare providers, by stratum

	Rural			Urban		
	n	%	CI	n	%	CI
**Child Age (in years)**	547			537		
0	102	18.6	[15.6-22.1]	102	19	[15.9-22.5]
1	115	21	[17.8-24.6]	121	22.5	[19.2-26.3]
2	115	21	[17.8-24.6]	107	19.9	[16.8-23.5]
3	109	19.9	[16.8-23.5]	100	18.6	[15.5-22.1]
4	106	19.4	[16.3-22.9]	107	19.9	[16.8-23.5]
**Child Sex**	547			537		
Female	274	50.1	[45.9-54.3]	277	51.6	[47.3-55.8]
Male	273	49.9	[45.7-54.1]	260	48.4	[44.2-52.7]
**Maternal Age (in years)**	387			451		
15-19	47	12.1	[9.2-15.8]	51	11.3	[8.7-14.6]
20-29	155	40.1	[35.3-45.0]	253	56.1	[51.5-60.6]
30-39	126	32.6	[28.1-37.4]	127	28.2	[24.2-32.5]
40-49	59	15.2	[12.0-19.2]	20	4.4	[2.9-6.8]
**Maternal Education**	387			451		
No education or primary incomplete	97	25.1	[21.0-29.6]	82	18.2	[14.9-22.0]
Primary complete	118	30.5	[26.1-35.3]	69	15.3	[12.3-18.9]
Secondary incomplete	138	35.7	[31.0-40.6]	171	37.9	[33.5-42.5]
Secondary complete or higher	34	8.8	[6.3-12.1]	129	28.6	[24.6-33.0]
**Providers participating in preparedness assessment**	54			29		
Govt hospital	0			1		
Govt health center/ post	5			2		
Govt CBA / fieldworker	19			9		
Pvt hospital / clinic	0			5		
Pharmacy	0			6		
Shop / market	1			3		
Traditional / faith-based practitioner	29			3		

Supplemental Table 2. Median structural quality domain scores by provider category (each category score out of one by provider)

Provider Category	Sample Size	Diagnostics		Basic Medicines		Severe / Complicated Illness Medicines		Human Resources		Management Capacity		Knowledge		Structural Quality Score	
		%	(IQR)	%	(IQR)	%	(IQR)	%	(IQR)	%	(IQR)	%	(IQR)	%	(IQR)
Govt hospital / health center / post	8	100	(83.3-100)	87.5	(75-100)	83.3	(66.7-100)	66.7	(33.3-66.7)	100	(100-100)	51.7	(47.1-64.3)	81.5	(74.8-86.6)
Govt CBA / fieldworker	28	100	(50-100)	66.7	(33.3-100)	-	-	66.7	(33.3-66.7)	40	(20-40)	100	(100-100)	70.7	(57.7-71.3)
Pvt hospital / clinic	5	80	(66.7-83.3)	100	(75-100)	66.7	(66.7-66.7)	33.3	(33.3-33.3)	100	(100-100)	54.3	(45.8-56.8)	68.7	(58.4-75.7)
Pharmacy	6	33.3	(0-50)	100	(75-100)	66.7	(33.3-66.7)	33.3	(0-66.7)	20	(0-60)	0	(0-78.6)	43.8	(33.3-61.3)
Shop / market	4	-	-	-	-	-	-	-	-	0	(0-0)	0	(0-0)	0	(0-0)
Traditional / faith-based	32	16.7	(0-50)	0	(0-0)	-	-	0	(0-0)	20	(0-20)	25	(0-50)	16.7	(5-28)

*Presented using collapsed provider categories to preserve confidentiality of providers. Categories defined in Box1 1 used in all linking analyses restricted by source of care provider category.

Supplemental Table 3. Variability of structural quality scores within provider category by HFCA, strata, and overall assessed as quartile coefficient of dispersion and ANOVA test of within versus between provider category variance

Provider Category	HFCA										Strata				Overall	
	RURAL A		RURAL B		RURAL C		URBAN A		URBAN B		Rural		Urban			
	n	Quartile COD	n	Quartile COD	n	Quartile COD	n	Quartile COD	n	Quartile COD	n	Quartile COD	n	Quartile COD	n	Quartile COD
Hospital	0	-	0	-	0	-	0	-	0	-	0	-	1	0	1	0
Govt health center / post	1	0	1	0	1	0	1	0	1	0	5	0.09	2	0.01	7	0.09
Govt CBA / fieldworker	7	0.11	7	0.16	5	0.21	4	0.06	5	0.07	19	0.12	9	0.06	28	0.11
Pvt hospital / clinic	0	-	0	-	0	-	2	0.09	3	0.29	0	-	5	0.13	5	0.13
Pharmacy	0	-	0	-	0	-	1	0	5	0.29	0	-	6	0.29	6	0.30
Shop / market	0	-	0	-	1	-	3	0	0	-	1	0	3	0	4	0
Traditional / faith-based	10	0.38	8	0.30	11	0.46	2	1	1	0	29	0.70	3	1	32	0.70
**ANOVA Test**																
Mean Square Between Group Variance	0.779		0.285		0.342		0.234		0.104		1.156		0.273		0.89	
Mean Square Within Group Variance	0.008		0.018		0.015		0.035		0.051		0.015		0.041		0.2	
F statistic, p	96.02, <0.001		16.05, <0.001		21.42, <0.001		6.60, 0.014		2.03, ns		73.18, <0.001		6.71, <0.001		40.28, <0.001	

Supplemental Table 4. Proportion of care-seeking events linked to original source of care by single-link method by provider category and stratum

**All Providers**						
**Nearest Absolute Distance**		**Rural**			**Urban**	
	# Original	# Linked	% Linked Original	# Original	# Linked	% Linked Original
Govt hospital	0	-	-	5	5	100
Govt health center / post	122	111	91.0%	111	100	90.1
Govt CBA / fieldworker	36	29	80.6	0*	-	-
Pvt hospital / clinic	0	-	-	1	0	0
Pharmacy	0*	-	-	2	1	50
Shop / market	2	2	100	1*	0	0
Traditional / faith-based	4*	4	100	0	-	-
All Sources of Care	164	146	89.0%	120	106	88.3%
**Nearest Road Distance**		**Rural**			**Urban**	
	# Original	# Linked	% Linked Original	# Original	# Linked	% Linked Original
Govt hospital	0	-	-	5	5	100
Govt health center / post	122	100	82.0%	111	86	77.5
Govt CBA / fieldworker	36	24	66.7	0*	-	-
Pvt hospital / clinic	0	-	-	1	0	0
Pharmacy	0*	-	-	2	0	0
Shop / market	2	2	100	1*	1	100
Traditional / faith-based	4*	2	50	0	-	-
All Sources of Care	164	128	78.0%	120	92	76.7%

**Facility-Based Providers Only**						
**Nearest Absolute Distance**		**Rural**			**Urban**	
	# Original	# Linked	% Linked Original	# Original	# Linked	% Linked Original
Govt hospital	0	-	-	5	5	100
Govt health center / post	122	111	91.0%	111	100	90.1
Govt CBA / fieldworker	36	0	0	0*	-	-
Pvt hospital / clinic	0	-	-	1	0	0
Pharmacy	0*	-	-	2	0	0
Shop / market	2	0	0	1*	0	0
Traditional / faith-based	4*	0	0	0	-	-
All Sources of Care	164	111	67.7%	120	106	88.3%
**Nearest Road Distance**		**Rural**			**Urban**	
	# Original	# Linked	% Linked Original	# Original	# Linked	% Linked Original
Govt hospital	0	-	-	5	5	100
Govt health center / post	122	100	82.0%	111	86	77.5
Govt CBA / fieldworker	36	0	0	0*	-	-
Pvt hospital / clinic	0	-	-	1	0	0
Pharmacy	0*	-	-	2	0	0
Shop / market	2	0	0	1*	0	0
Traditional / faith-based	4*	0	0	0	-	-
All Sources of Care	164	100	61.0%	120	91	75.8%

Supplemental Table 5. Proportion of children that were linked to any provider, by provider category and stratum

All Providers

**Radius - 5 km**	**Rural**			**Urban**		
	# Original	# Matched	% Matched	# Original	# Matched	% Matched
Govt hospital	0	-	-	5	5	100%
Govt health center / post	122	65	53%	111	111	100%
Govt CBA / fieldworker	36	36	100%	1	1	100%
Pvt hospital / clinic	0	-	-	1	1	100%
Pharmacy	1	0	0%	2	2	100%
Shop / market	2	2	100%	9	9	100%
Traditional / faith-based	5	3	60.0%	0	-	-
All Sources of Care	166	106	63.8%	129	129	100%
**Administrative Unit - HFCA**	**Rural**			**Urban**		
	# Original	# Matched	% Matched	# Original	# Matched	% Matched
Govt hospital	0	-		5	5	100%
Govt health center / post	122	122	100%	111	111	100%
Govt CBA / fieldworker	36	36	100%	1	1	100%
Pvt hospital / clinic	0	-		1	1	100%
Pharmacy	1	0	0%	2	2	100%
Shop / market	2	2	100%	9	9	100%
Traditional / faith-based	5	5	100%	0	-	-
All Sources of Care	166	165	99.4%	129	129	100%
**Administrative Unit - Total Area**	**Rural**			**Urban**		
	# Original	# Matched	% Matched	# Original	# Matched	% Matched
Govt hospital	0	-		5	5	100%
Govt health center / post	122	122	100%	111	111	100%
Govt CBA / fieldworker	36	36	100%	1	1	100%
Pvt hospital / clinic	0	-		1	1	100%
Pharmacy	1	1	100%	2	2	100%
Shop / market	2	2	100%	9	9	100%
Traditional / faith-based	5	5	100%	0	-	-
All Sources of Care	166	165	100%	129	129	100%

Facility-Based Providers Only

**Radius - 5 km**	**Rural**			**Urban**		
	# Original	# Matched	% Matched	# Original	# Matched	% Matched
Govt hospital	0	-	-	5	5	100%
Govt health center / post	122	65	53%	111	111	100%
Govt CBA / fieldworker	36	0	0%	1	0	0%
Pvt hospital / clinic	0	-	-	1	1	100%
Pharmacy	1	0	0%	2	0	0%
Shop / market	2	0	0%	9	0	0%
Traditional / faith-based	5	0	0%	0	-	-
All Sources of Care	166	65	39%	129	117	91%
**Administrative Unit - HFCA**	**Rural**			**Urban**		
	# Original	# Matched	% Matched	# Original	# Matched	% Matched
Govt hospital	0	-		5	5	100%
Govt health center / post	122	122	100%	111	111	100%
Govt CBA / fieldworker	36	0	0%	1	0	0%
Pvt hospital / clinic	0	-		1	1	100%
Pharmacy	1	0	0%	2	0	0%
Shop / market	2	0	0%	9	0	0%
Traditional / faith-based	5	0	0%	0	-	-
All Sources of Care	166	122	73.5%	129	117	91%
**Administrative Unit -**	**Rural**			**Urban**		

**Total Area**	# Original	# Matched	% Matched	# Original	# Matched	% Matched
Govt hospital	0	-		5	5	100%
Govt health center / post	122	122	100%	111	111	100%
Govt CBA / fieldworker	36	0	0%	1	0	0%
Pvt hospital / clinic	0	-		1	1	100%
Pharmacy	1	0	0%	2	0	0%
Shop / market	2	0	0%	9	0	0%
Traditional / faith-based	5	0	0%	0	-	-
All Sources of Care	166	122	73.5%	129	117	91%

Supplemental Table 6. Distance traveled from home to reported source of care by provider category

Provider category		# Linked	Mean	SD	Min	Max
Govt hospital		5	2.80	0.31	2.36	3.21
Govt health center / post	Urban	113	1.63	2.42	0.10	19.04*
	Rural	120	5.41	3.81	0.32	16.13
Govt CBA / fieldworker	Urban	0	-	-	-	-
	Rural	36	2.04	1.34	0.01	5.37
Pvt hospital / clinic		1	4.39	-	4.39	4.39
Pharmacy		2	1.84	0.25	1.67	2.02
Shop / market	Urban	1	1.93	-	1.93	1.93
	Rural	2	2.90	1.64	1.73	4.07
Traditional / faith-based practitioner	Urban	0	-	-	-	-
	Rural	4	3.05	3.48	0.03	6.07

*2 rural children sought care from an urban facility

Supplemental Table 7. Average number of provider links among children that linked to source of care using geolinking method, by provider category and stratum

**Radius - 5 km**	Rural		Urban	
	Mean	[Range]	Mean	[Range]
Govt hospital	-	-	1	[1-1]
Govt health center / post	1	[1-1]	2	[2-2]
Govt CBA / fieldworker	2.03	[1-4]	5	[5-5]
Pvt hospital / clinic	-	-	5	[5-5]
Pharmacy	-	-	6	[6-6]
Shop / market	1	[1-1]	3	[3-3]
Traditional / faith-based	3.33	[2-4]	-	-
All Sources of Care	1.44	[1-5]	2.2	[1-8]

**Administrative Unit - HFCA**	Rural		Urban	
	Mean	[Range]	Mean	[Range]
Govt hospital	-	-	1	[1-1]
Govt health center / post	1	[1-1]	1	[1-1]
Govt CBA / fieldworker	6.11	[5-7]	4	[4-4]
Pvt hospital / clinic	-	-	2	[2-2]
Pharmacy	-	-	5	[5-5]
Shop / market	1	[1-1]	3	[3-3]
Traditional / faith-based	9.8	[8-11]	-	-
All Sources of Care	2.51	2.51 [1-11]	1.28	1.28 [1-6]

**Administrative Unit - Total Area**	Rural		Urban	
	Mean	[Range]	Mean	[Range]
Govt hospital	-	-	1	[1-1]
Govt health center / post	7	[7-7]	7	[7-7]
Govt CBA / fieldworker	28	[28-28]	28	[28-28]
Pvt hospital / clinic	-	-	5	[5-5]
Pharmacy	6	[6-6]	6	[6-6]
Shop / market	4	[4-4]	4	[4-4]
Traditional / faith-based	32	[32-32]	-	-
All Sources of Care	12.97 [4-39]	12.97 [4-39]	6.96 [1-28]	6.96 [1-28]

Supplemental Table 8. Source of care by provider category, modeled through KDE single link and KDE weighted link methods, by provider category and stratum

**All Providers**				
**KDE - Single Link**	**Rural**		**Urban**	
	# linked	% children linked	# linked	% children linked
	199		186	
Govt hospital	0	0%	34	18.3%
Govt health center / post	74	37.2%	0	0%
Govt CBA / fieldworker	65	32.7%	66	35.5%
Pvt hospital / clinic	0	0%	57	30.6%
Pharmacy	0	0%	29	15.6%
Shop / market	0	0%	0	0%
Traditional / faith-based	20	10.1%	0	0%
No Source	40	20.1%	0	0%
**KDE - Weighted Link**	**Rural**		**Urban**	
	# linked	% children linked	# linked	% children linked
	199		186	
Govt hospital	6	3%	186	100%
Govt health center / post	92	46.2%	186	100%
Govt CBA / fieldworker	69	34.7%	121	65.1%
Pvt hospital / clinic	0	0%	186	100%
Pharmacy	0	0%	150	80.6%
Shop / market	0	0%	0	0%
Traditional / faith-based	35	17.6%	85	45.7%
No Source	40	20.1%	0	0%

**Facility-Based Providers Only**				
**KDE - Single Link**	**Rural**		**Urban**	
	# linked	% children linked	# linked	% children linked
	199		186	
Govt hospital	3	1.5%	34	18.3%
Govt health center / post	89	44.7%	0	0%
Govt CBA / fieldworker	0	0%	0	0%
Pvt hospital / clinic	0	0%	152	81.7%
Pharmacy	0	0%	0	0%
Shop / market	0	0%	0	0%
Traditional / faith-based	0	0%	0	0%
No Source	107	53.8%	0	0%
**KDE - Weighted Link**	**Rural**		**Urban**	
	# linked	% children linked	# linked	% children linked
	199		186	
Govt hospital	6	3%	186	100%
Govt health center / post	92	46.2%	186	100%
Govt CBA / fieldworker	0	0%	0	0%
Pvt hospital / clinic	0	0%	186	100%
Pharmacy	0	0%	0	0%
Shop / market	0	0%	0	0%
Traditional / faith-based	0	0%	0	0%
No Source	107	53.8%	0	0%

Supplemental Table 9. Effective coverage of management of child illness and difference in estimate from the exact-match all provider coverage, by linking method and stratum using primary and alternative assumptions for missing links

A. All Providers

Linking Method	Unlinked care-seeking event assigned:	Rural				Urban			
		%	[95% CI]	Diff	Sign	%	[95% CI]	Diff	Sign
**Exact-Match**		60.3	[55.6 - 65.1]	REF		49	[43.6 - 54.5]	REF	
**Single Match**									
Nearest-Absolute Distance	Average	61.1	[56.3 - 65.9]	0.8	ns	49.1	[43.7 - 54.6]	0.1	ns
	Zero	61.1	[56.3 - 65.9]	0.8	ns	49.1	[43.7 - 54.6]	0.1	ns
Nearest-Road Distance	Average	58.8	[54.1 - 63.5]	-1.5	ns	48.7	[43.2 - 54.1]	-0.3	ns
	Zero	58.8	[54.1 - 63.5]	-1.5	ns	48.7	[43.2 - 54.1]	-0.3	ns
**Aggregate Match**									
Radius-5 km	Average	59.4	[54.8 - 64.1]	-0.9	ns	49.2	[43.7 - 54.7]	0.2	ns
	Zero	38.8	[33.4 - 44.2]	-21.5	***	49.2	[43.7 - 54.7]	0.2	ns
Administrative unit-HFCA	Average	59.8	[55.1 - 64.5]	-0.5	ns	49.1	[43.6 - 54.6]	0.1	ns
	Zero	59.5	[54.8 - 64.3]	-0.8	ns	49	[43.6 - 54.5]	0	ns
Administrative unit-Total Area	Average	57.9	[53.4 - 62.4]	-2.4	ns	49.4	[43.9 - 54.9]	0.4	ns
	Zero	57.9	[53.4 - 62.4]	-2.4	ns	49.4	[43.9 - 54.9]	0.4	ns
**KDE**									
Single Highest		55	[50.4 - 59.6]	-5.3	*	71.8	[69.3 - 74.2]	22.8	***
Weighted Aggregate		54.9	[50.4 - 59.5]	-5.4	*	74.3	[73.2 - 75.5]	25.3	***

B. Facility-Based Providers Only

	Unlinked care-seeking event assigned:	Care-seeking with CBA linked to:	Rural				Urban			
			%	[95% CI]	Diff	Sign	%	[95% CI]	Diff	Sign
**Exact-Match**	Average	Govt HC	62.1	[57.1 - 67.0]	1.8	ns	48.7	[43.2 - 54.2]	-0.3	ns
	Zero	No Source	50.2	[44.6 - 55.8]	-10.1	***	48.4	[42.8 - 53.9]	-0.6	ns
**Single Match**										
Nearest-Absolute Distance	Average	Govt HC	62.6	[57.6 - 67.6]	2.3	ns	48.7	[43.2 - 54.2]	-0.3	ns
	Zero	No Source	51.5	[45.8 - 57.3]	-8.8	**	48.9	[43.4 - 54.4]	-0.1	ns
Nearest-Road Distance	Average	Govt HC	61	[56.2 - 65.9]	0.7	ns	48.6	[43.1 - 54.0]	-0.4	ns
	Zero	No Source	49.9	[44.3 - 55.4]	-10.4	***	48.8	[43.2 - 54.3]	-0.2	ns
**Aggregate Match**										
Radius-5 km	Average	Govt HC	61.2	[56.4 - 66.1]	0.9	ns	48.9	[43.4 - 54.5]	-0.1	ns
	Zero	No Source	27.3	[21.8 - 32.7]	-33	***	49.2	[43.6 - 54.7]	0.2	ns
Administrative unit-HFCA	Average	Govt HC	62.8	[57.8 - 67.8]	2.5	ns	48.8	[43.3 - 54.3]	-0.2	ns
	Zero	No Source	51	[45.3 - 56.7]	-9.3	**	48.9	[43.4 - 54.5]	-0.1	ns
Administrative unit-Total Area	Average	Govt HC	59.9	[55.2 - 64.6]	-0.4	ns	49	[43.5 - 54.6]	0	ns
	Zero	No Source	48.7	[43.3 - 54.1]	-11.6	***	49.3	[43.7 - 54.9]	0.3	ns
**KDE**										
Single Highest			38.6	[32.8 - 44.4]	-21.7	***	79	[77.8 - 80.3]	30	***
Weighted Aggregate			38.6	[32.8 - 44.4]	-21.7	***	82.4	[81.9 - 82.9]	33.4	***
